# Diseases of the Spine

**Published:** 1880-12

**Authors:** 


### DISEASES OF THE SPINE.

In every community a certain number of young persons, a majority of whom are young women, have spinal troubles, ranging from a slight weakness of the back to actual destruction of one or more of the spinal vertebræ.

In the latter case all treatment is simply palliative, since nothing can repair what has been actually destroyed; but all ordinary cases of "spinal irritation" may be cured in six weeks to six months by proper treatment. I will not stop to refer to the causes of this complaint: they are many; some preventable, others, perhaps, may not be. The main symptoms are severe headache and pain in the back, with tenderness of the spine on pressure, attacks of nervousness, alternately crying and laughing, loss of appetite and more or less debility.

This is an irritation of the spinal cord, and the object is to relieve it by drawing the irritation to the surface and building up the general health. I have employed various counter-irritants for this purpose, but none have answered so well as the tartar emetic ointment. This should be rubbed on the spine every day until a thick crop of sores is produced, and, as they heal, rub between the sores to bring out a fresh crop of them. This treatment should be continued until every unfavorable symptom disappears; if it takes a year.

During the treatment it may be well to give tonics, but I have usually found good nutritious food quite sufficient. Above all the bowels must be kept regular and in a healthy condition. This class of patients are too often "patched up" with tonics only, and left to their fate, which is frequently a sad one, since they are not only deprived of many of the pleasures of life, but have their full share of its pains. To add to their troubles, they seldom have the sympathy of their friends, who are generally more inclined to censure than to pity for what they wrongly suppose to be their unpleasant peculiarities.

There are two diseases—for it is a mistake to call them simply "derangements of the system"—that are usually neglected, because they are not regarded as immediately dangerous; these are "Spinal Irritation" and "Constipation." There is not, perhaps, one case in a hundred of either of these diseases that may not be permanently and radically cured in three months with proper treatment. The trouble is that most men look after their health after they have attended to everything else, and it may then be too late.

